# Comparative analysis of the *SPL* gene family in five Rosaceae species: *Fragaria vesca*, *Malus domestica*, *Prunus persica*, *Rubus occidentalis*, and *Pyrus pyrifolia*

**DOI:** 10.1515/biol-2021-0020

**Published:** 2021-02-19

**Authors:** Xuwen Jiang, Peng Chen, Xiaowen Zhang, Qizhi Liu, Heqin Li

**Affiliations:** Dryland Technology Key Laboratory of Shandong Province, College of Agronomy, Qingdao Agricultural University, Changcheng Road No. 700, Chengyang District, Qingdao, 266109, Shandong, China; Department of Entomology, College of plant protection, China Agricultural University, Yuanmingyuan West Road No. 2, Haidian District, Beijing, 100193, China; Institute of Plant Protection, Shandong Academy of Agricultural Sciences, Gongye North Road No. 202, Jinan, 250100, China

**Keywords:** *SPL* gene family, phylogenetic analysis, miR156, functional divergence, Rosaceae species

## Abstract

SQUAMOSA promoter-binding protein-like (SPL) transcription factors are very important for the plant growth and development. Here 15 *RoSPLs* were identified in *Rubus occidentalis*. The conserved domains and motifs, phylogenetic relationships, posttranscriptional regulation, and physiological function of the 92 *SPL* family genes in *Fragaria vesca*, *Malus domestica*, *Prunus persica*, *R. occidentalis*, and *Pyrus pyrifolia* were analyzed. Sequence alignment and phylogenetic analysis showed the SPL proteins had sequence conservation, some FvSPLs could be lost or developed, and there was a closer relationship between *M. domestica* and *P. pyrifolia*, *F. vesca* and *R. occidentalis*, respectively. Genes with similar motifs clustering together in the same group had their functional redundancy. Based on the function of *SPLs* in *Arabidopsis thaliana*, these *SPLs* could be involved in vegetative transition from juvenile to adult, morphological change in the reproductive phase, anthocyanin biosynthesis, and defense stress. Forty-eight *SPLs* had complementary sequences of miR156, of which nine *PrpSPLs* in *P. persica* and eight *RoSPLs* in *R. occidentalis* as the potential targets of miR156 were reported for the first time, suggesting the conservative regulatory effects of miR156 and indicating the roles of miR156-SPL modules in plant growth, development, and defense response. It provides a basic understanding of *SPLs* in Rosaceae plants.

## 1 Introduction

SQUAMOSA promoter-binding protein-like (SPL) gene encodes plant-specific transcription factors in all green plants and cannot be found in prokaryotes, fungi, or animals. SPL protein contains the very conservative DNA-binding domain, namely the squamosa promoter binding protein (SBP) domain, consisting of ∼76 amino acid (aa) residues to carry out sequence-specific DNA binding and nuclear localization [1]. The SBP domain was composed of two zinc-binding sites (Cys–Cys–Cys–His and Cys–Cys–His–Cys) and a nuclear localization signal (NLS) partially overlapping with Cys–Cys–His–Cys [2]. Since 1996, *SPL* genes have been reported in many plants, such as *Arabidopsis thaliana* [3], rice [4], maize [5], *Petunia* [6], *Dichanthelium oligosanthes* [1], tea [7], *Jatropha curcas*[8], apple [9], pear [10], peach, and strawberry [11]. Plant genomes show remarkable variation in size and organization, as well as the number of *SPLs*. For example, 16 *SPLs* are identified in *Arabidopsis* genome [3], whereas 32 *SPLs* are present in the maize genome [5].

*SPL* genes are very important for plant growth and development, as they regulate the specific downstream gene expression, such as the phase transition from vegetative to reproductive, trichome development, leaf development, fruit ripening, pollen sac development, fertility, plant hormone signaling [11,12,13], toxin resistance [14], copper deficiency response [15], temperature, salinity, and drought stress tolerance [7,16]. In addition, *SPLs* are targeted by miR156 family members [17]. Their importance has been reported in multiple plant developmental processes. For example, in maize and *Arabidopsis*, the expression level of *SPLs* is reduced by *miR156*, which prolongs the juvenile phase in the miR156 mutation [18,19]; in rice, *OsmiR156b* or *OsmiR156h* regulates flowering time [4]; in *Arabidopsis*, overexpressing *miR156* produces more lateral roots by repressing at least one representative from the *SPL3*, *SPL9*, and *SPL10* [20]. The biosynthesis of anthocyanin in *Arabidopsis* is regulated by at least one miR156-targeted *SPL* factor, such as *SPL9* [21]. These studies suggest the physiological significance and diversity of some *SPLs* in *Arabidopsis* and other model plants. However, the functions of most *SPLs* are needed to be investigated further in other plants.

The Rosaceae family includes many famous fruit-producing plant species, such as *Malus*, *Pyrus*, *Prunus*, *Fragaria*, and *Rubus* [22]. Thanks to the development of genome sequencing technology, genomes of some of these plants have been already published. This provides basic data for studying important functional genes at the genome-wide level. Previous studies showed that strawberry, apple, peach, and pear genomes have 14, 27, 17, and 19 *SPLs*, respectively [9,10,11,23]. However, no information about *SPLs* are available in black raspberry (*Rubus occidentalis*), a species of raspberry in the Rosaceae family. Here we identified the *SPL* genes in black raspberry and carried out phylogenetic analysis to study the evolution and function of *SPL* gene family in *Fragaria vesca* (strawberry), *Malus domestica* (apple), *Prunus persica* (peach), *R. occidentalis* (black raspberry), and *Pyrus pyrifolia* (pear) genomes. The feature of the conserved domain and the conserved motifs, the function of the miR156 target, and functional diversity of *SPLs* are also discussed. The results will provide an important theoretical basis for further exploring *SPL* family gene in Rosaceae plant species.

## 2 Materials and methods

### 2.1 *SPL* gene sequences in strawberry, apple, peach, black raspberry, and pear

First, the nucleotide and protein sequences of SPLs from *Arabidopsis*, *F. vesca*, *M. domestica*, and *P. persica* were obtained from the comparative genome database Phytozome v12.1 (https://phytozome.jgi.doe.gov/pz/portal.html). For *P. pyrifolia*, the sequences of *SPLs* were collected according to Qian et al. [10]. For *R. occidentalis*, the sequences of genome, coding sequence (CDS), and protein were obtained from GENOME DATABASE FOR ROSACEAE (https://www.rosaceae.org). Then, the analysis of AtSPL proteins against the downloaded CDS sequences of *R. occidentalis* was performed with tBLASTn with the *E*-value ≤ *e* ^−10^. According to the alignments between the candidate DNA sequences and SPL proteins from other plant species, gene models of *RoSPLs* were predicted via BLASTx.

### 2.2 Identification of conserved domains and motifs of SPL proteins

Conserved Domain Database (CDD, http://www.ncbi.nlm.nih.gov/Structure/cdd/wrpsb.cgi) was used to search for the SBP domain from all candidate protein sequences. Sequences containing the SBP domain were considered as *SPL* genes. The domain alignment was carried out using DNAMAN8 software (https://www.lynnon.com/). Sequence logos were created through WebLogo (http://weblogo.berkeley.edu/logo.cgi). Potential protein motifs were predicted by the MEME package (http://meme.sdsc.edu/meme/) with parameters as follows: zero and one per sequence, 20 as the maximum number of motifs and the minimum width of motif, and 160 as the maximum width of motif, an *E*-value ≤ *e* ^*−*10^.

### 2.3 SPL protein sequence alignment and phylogenetic analysis

ClustalW in MEGA5.1 software (http://www.megasoftware.net) was used to align the full-length SPL protein sequences, and neighbor-joining method with *P*-distance model and 1,000 bootstrap values (>50%) in MEGA5.1 were used to construct the phylogenetic relationships.

### 2.4 Prediction of miR156 target genes

All mature sequences of miR156 from strawberry, apple, peach, and pear were obtained according to the previous reports [24,25,26,27]. For black raspberry, the mature sequences of miR156 genes were obtained according to ath-miR156 sequences against the downloaded genomic sequences of *R. occidentalis* using tBLASTn. Target sites in *SPL* genes were obtained in the five Rosaceae plants through the online psRNATarget server (http://plantgrn.noble.org/psRNATarget/) with default settings. The maximum expectation is 3.0, and the target site accessibility evaluation by calculating unpaired energy is 40.

## 3 Results

### 3.1 Genome-wide identification of *RoSPL* genes

Based on the genome of *R. occidentalis*, 15 *SPL* genes were identified and named from *RoSPL1* to *RoSPL15* with GenBank accession number MN245039–MN245053 (Table 1). The number of *RoSPLs* is similar to *A. thaliana* (16), rice (19), and *D. oligosanthes* (14), suggesting that similar duplication events of *SPLs* were present in these plant species. The predicted RoSPL proteins varied from 17.9 kDa (RoSPL13) to 221.233 kDa (RoSPL1) in molecular weight, from 157 aa (RoSPL13) to 1996 aa (RoSPL1) in amino acid length, and from 5.98 (RoSPL2) to 9.22 (RoSPL10) in isoelectric point.

**Table 1:** Sequence features of the *SPL* gene family members in *R. occidentalis*

Gene name	GenBank accession	Scaffold	ORF (bp)	aa Length	*p*I	*M*_w_ (kDa)
*RoSPL1*	MN245039	Ro04_G21525	5,991	1,996	6.2	221.233
*RoSPL2*	MN245040	Ro04_G21525	3,048	1,015	5.98	112.195
*RoSPL3*	MN245041	Ro04_G07125	1,257	418	8	46.06
*RoSPL4*	MN245042	Ro03_G21167	594	197	8.93	21.996
*RoSPL5*	MN245043	Ro04_G02526	1,569	522	6.87	58.733
*RoSPL6*	MN245044	Ro05_G13862	3,225	1,074	8.71	119.015
*RoSPL7*	MN245045	Ro05_G03364	1,719	572	7.6	63.122
*RoSPL8*	MN245046	Ro04_G06346	2,463	820	6.4	91.644
*RoSPL9*	MN245047	Ro07_G24567	1,080	359	8.81	40.183
*RoSPL10*	MN245048	Ro06_G18927	1,128	375	9.22	40.209
*RoSPL11*	MN245049	Ro05_G01905	1,260	419	8.46	46.085
*RoSPL12*	MN245050	Ro04_G07111	915	304	8.81	33.554
*RoSPL13*	MN245051	Ro03_G33114	474	157	7.62	17.9
*RoSPL14*	MN245052	Ro06_G03784	1,206	401	8.97	44.863
*RoSPL15*	MN245053	Ro07_G24568	1,380	459	7.98	51.313

*Note*: ORF, open reading frame; aa, amino acids; *p*I, theoretical isoelectric point; *M*_w_, molecular weight.

### 3.2 Phylogenetic relationships of *SPL* genes in strawberry, apple, peach, black raspberry, and pear

**Figure 1:**
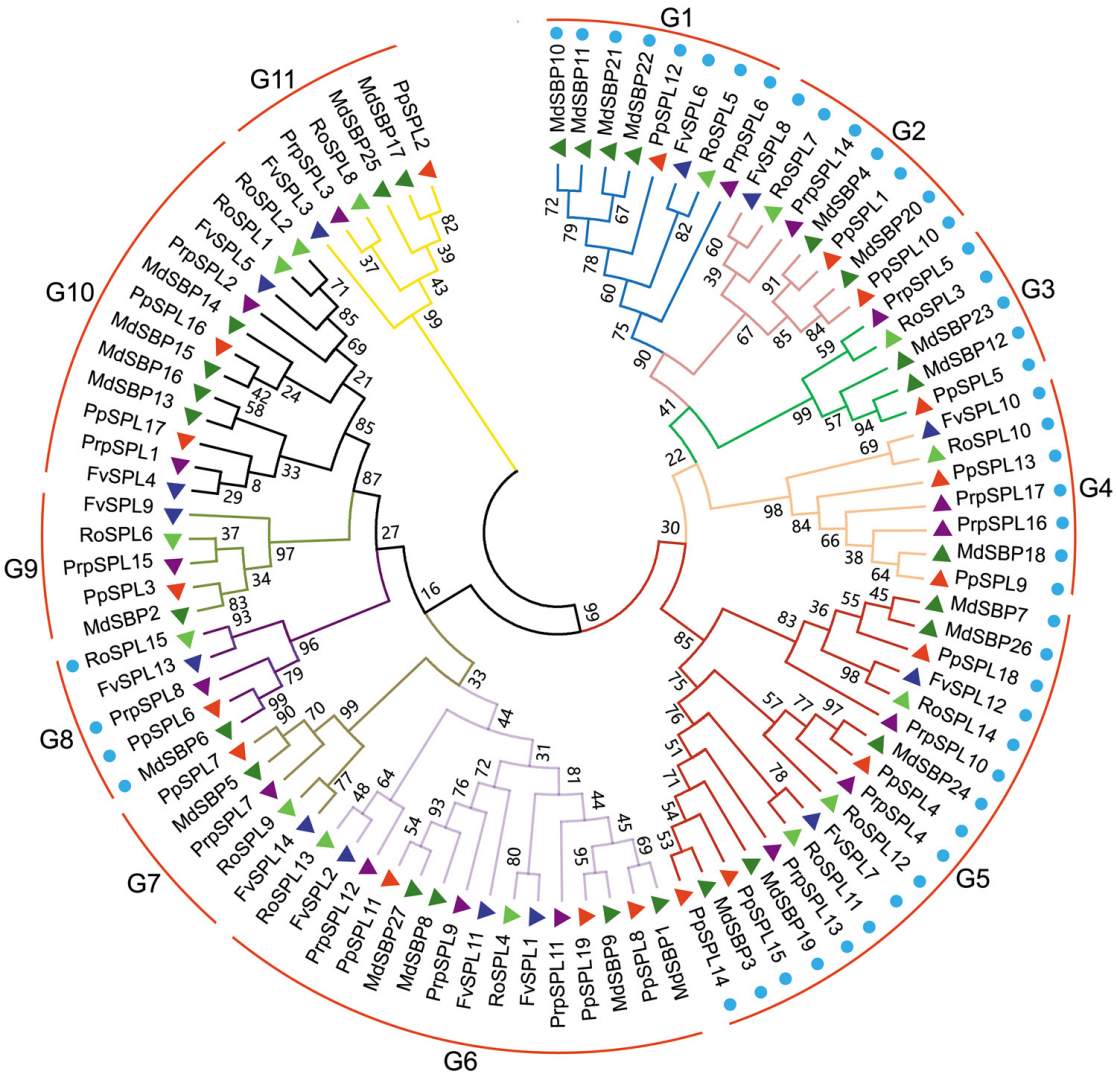
Phylogenetic analysis of *SPL* gene family in strawberry (*FvSPL*), apple (*MdSBP*), peach(*PrpSPL*), black raspberry (*RoSPL*), and pear (*PpSPL*). *SPL* genes containing complementary sequences to mature miR156 are marked with blue dots. MEGA5.1 with neighbor-joining (NJ) method was used to construct the phylogenetic tree.

Phylogenetic construction of the full-length sequences of SPL proteins in the five species analyzed from Rosaceae family (Table S1) resulted in generally similar topologies. The 92 proteins were classified into 11 groups (Figure 1). Except for the G3 group, all groups had at least one SPL protein from the five Rosaceae species, indicating the conservation of SPLs across Rosaceae genomes. However, the numbers of SPLs in certain groups were distinct between species, indicating the diversity of SPLs in Rosaceae family. The numbers of SPL proteins in G5, G6, and G10 groups were greater than those in other groups, with a large portion of SPLs found in apple (Figure 1). G1 group with eight SPL proteins had three SPL proteins from apple as G6 and G10 groups, and one SPL protein from strawberry, peach, black raspberry, and pear, respectively. It indicated that these groups have undergone extensive expansion after the speciation of apple. G2 and G4 groups each contained seven SPL proteins, with the same number of proteins from strawberry, black raspberry, and pear, but varying number of proteins from apple and peach. G11 group with six SPL proteins had two proteins from apple and one from peach, pear, black raspberry, and strawberry, respectively. G3, G7, G8, and G9 groups were all composed of five proteins, with the smallest number of SPL proteins. In G7, G8 and G9 groups the number of proteins from strawberry:apple:peach:black raspberry:pear was distributed as 1:1:1:1:1, indicating that these SPLs belong to orthologous proteins. Except for G3 group, all SPL proteins in other groups existed in the form of homologous proteins with each member from the five Rosaceae plants, indicating that these SPLs from different plant species shared common ancestors. FvSPLs were missing in the G3 group, indicating that some FvSPLs may be lost or may be developed in Rosaceae species. In total, 32 homologous groups were identified (Table S2). According to the phylogenetic relationship, PpSPL2 and MdSBP17, RoSPL8 and PrpSPL3 from the G11 group were highly probable to be orthologous proteins (Figure 1). Moreover, the other 30 pairs of SPLs from G1 to G10 groups seemed to be orthologous proteins (Figure 1 and Table S2). It suggested that these proteins may have close genetic relationship and play similar roles to their pairwise protein in the same group. Among the 32 pairs of SPLs, 13 pairs of PpSPL/MdSBP and 9 pairs of RoSPL/FvSPL were found, indicating that there was a closer relationship between apple and pear and strawberry and black raspberry, respectively. The results support the classification of plants, where pear and apple are Maloideae in the family Rosaceae, and strawberry and black raspberry are Rosoideae Focke in the family Rosaceae.

**Figure 2:**
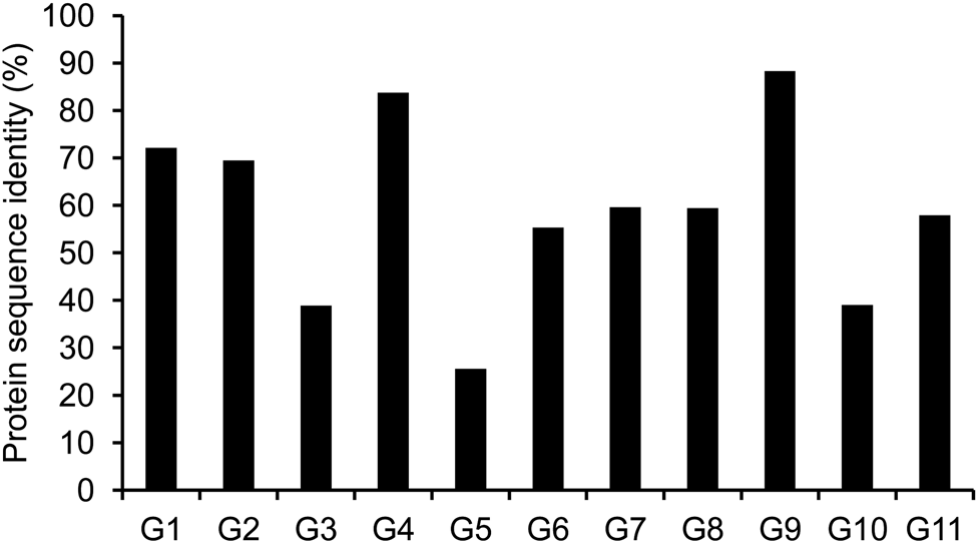
Analysis of protein sequence identity.

Moreover, homologous comparisons of the 92 full-length SPL protein sequences revealed genes with more than 50% protein sequence identity in G1, G2, G4, G6, G7, G8, G9, and G11 groups, and genes with more than 25% protein sequence identity in G3, G5, and G10 groups (Figure 2). These comparison analyses suggested the closer evolutionary relationship of SPLs in G1, G2, G4, G6, G7, G8, G9, and G11 groups.

### 3.3 Analysis of conserved domains and motifs in strawberry, apple, peach, black raspberry, and pear

**Figure 3:**
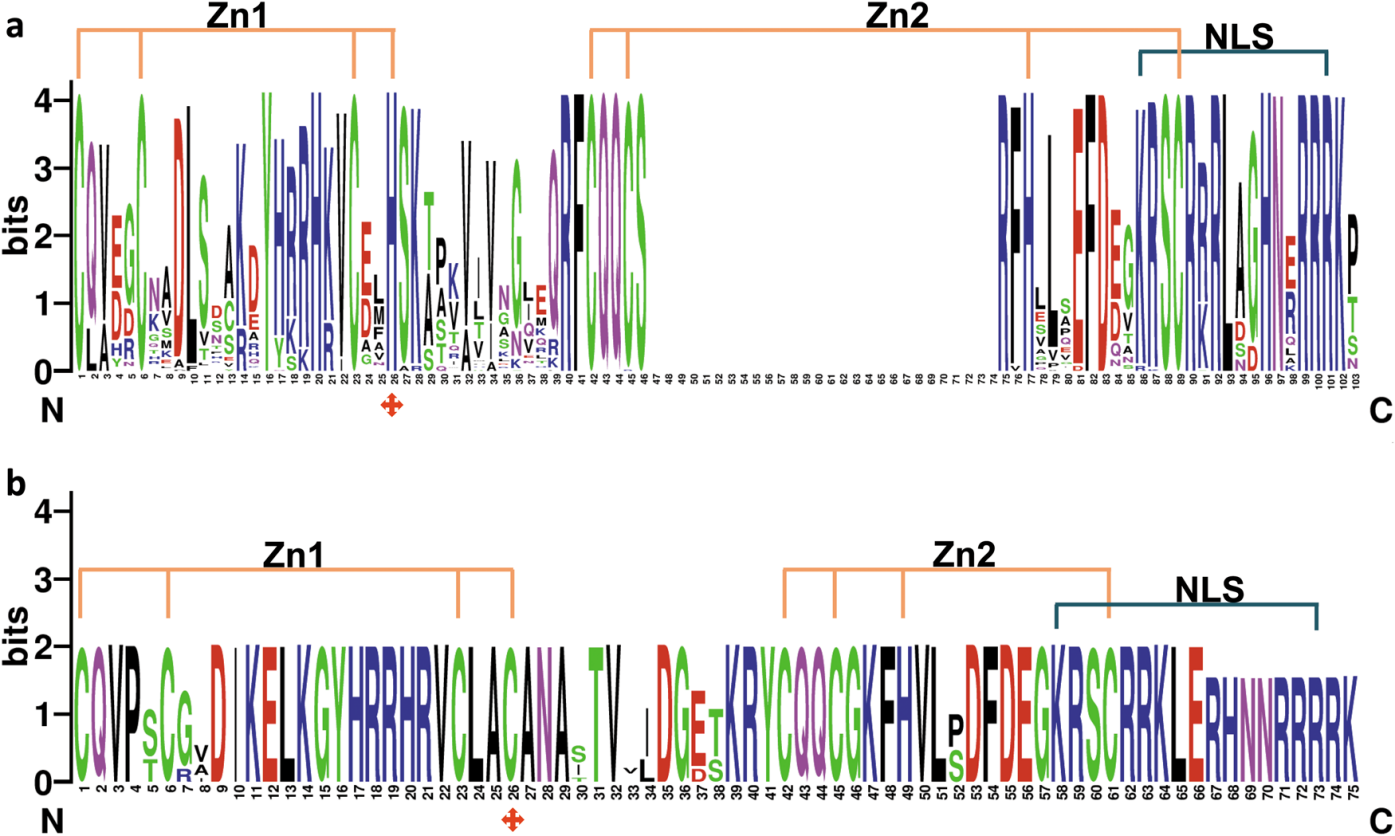
Sequence logo of the SBP domain of SPLs in strawberry, apple, peach, black raspberry, and pear. Sequence logos were created through WebLogo (http://weblogo.berkeley.edu/logo.cgi). (a) Sequence logo of the SBP domain of SPLs in G1–G10 groups; (b) sequence logo of the SBP domain of SPLs in the G11 group. Two conserved Zn-finger structures and the NLS are indicated.

**Figure 4:**
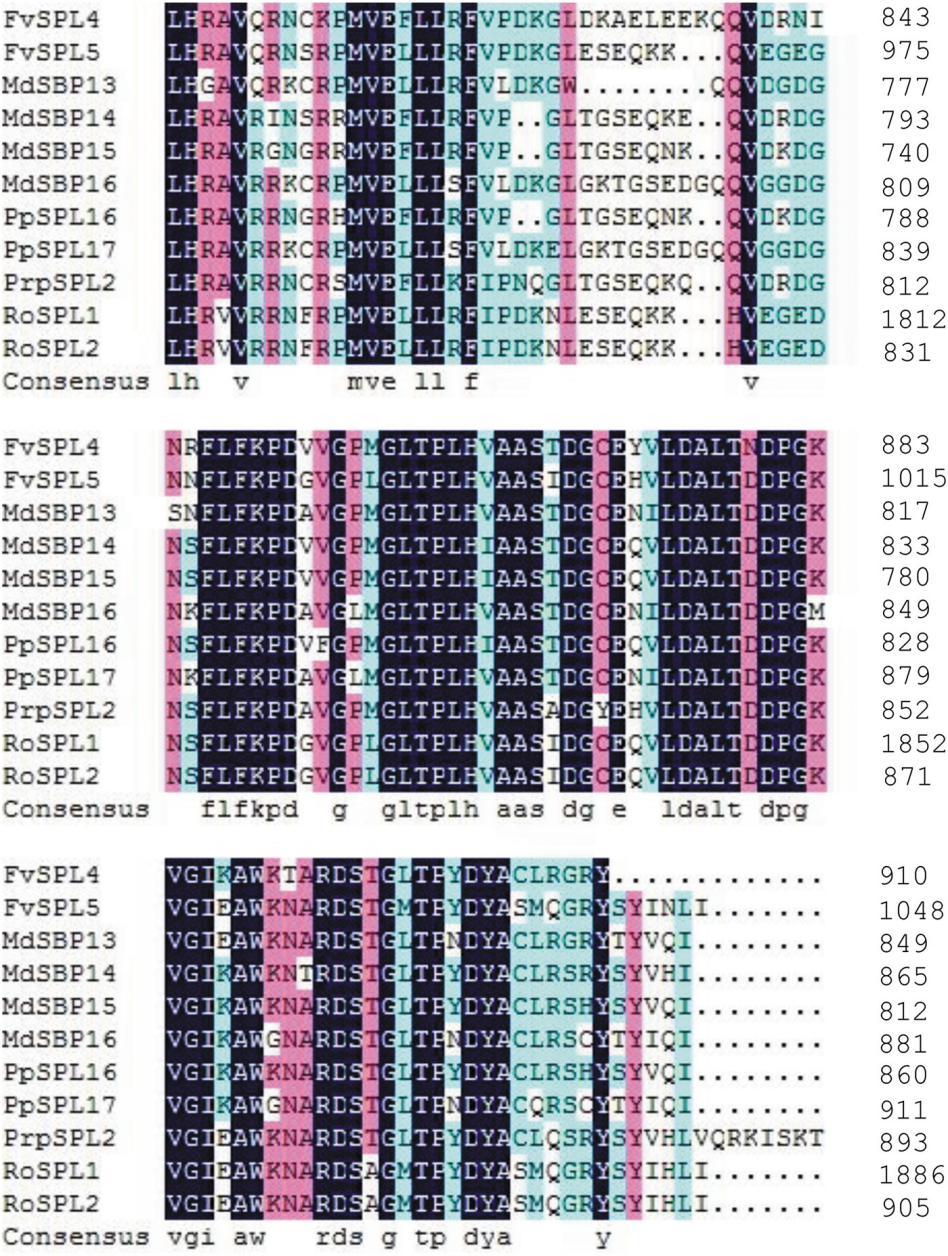
Alignment of the ANK-2 domain. The domain alignment was carried out using DNAMAN8. The ANK-2 domain is the result of the analysis of the SBP domain in CDD (http://www.ncbi.nlm.nih.gov/Structure/cdd/wrpsb.cgi) and is indicated by solid lines.

The SBP domain of the 92 SPLs was shown through sequence analysis (Figure 3). The Zn1 and Zn2 were shown in the SBP domain. Zn1 is Cys3His-type (CCCH-type) in SPLs from G1 to G10 groups (Figure 3a); however, the His residue in Zn1 is changed into a Cys residue in the G11 group, which resulted in the CCCH change into CCCC in the G11 group (Figure 3b). Compared with Zn1, the C2HC of Zn2 is very conservative in all SPLs analyzed. Not only Zn1 and Zn2, in the C-terminus, the SBP domain contains a conservative NLS overlapping with Zn2 (Figure 3). These results indicated that the domain organization has been constructed in the five Rosaceae species. These SBP domain locations, Zn1 and Zn2 binding sites, and NLS site were considered to be significant for specific recognition and binding to *cis*-elements in the promoter of nuclear genes [1,2]. Moreover, 11 SPLs belonging to the G10 group contain an ANK-2 domain (Figure 4), which is associated with protein–protein interaction in plant cells [28]. This indicated that it was valuable for interacting with other proteins for the role of SPLs in the same group.

**Figure 5:**
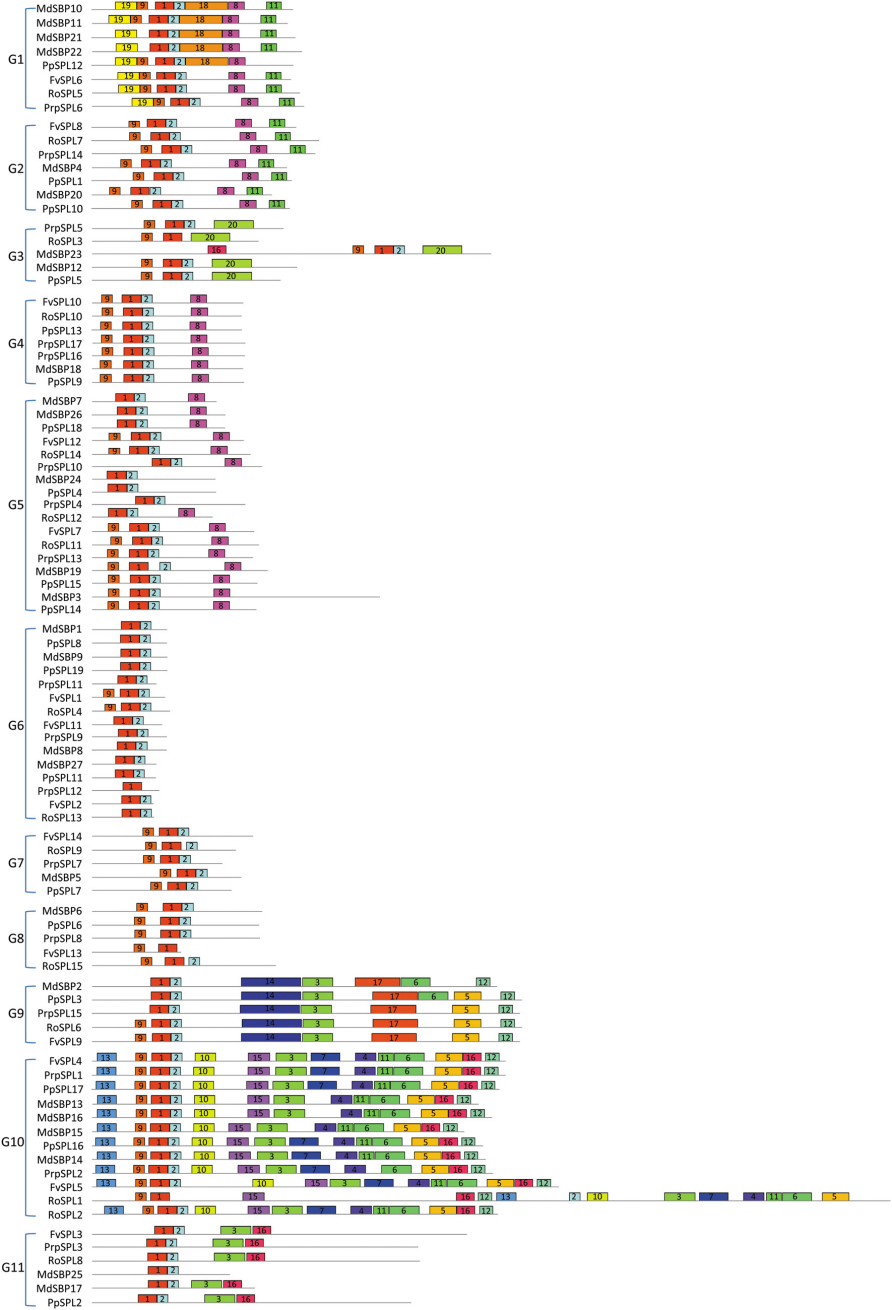
Distribution of conserved motifs in SPLs. Motifs represented with boxes are predicted using MEME. The number in boxes (1–20) represents motif 1 to motif 20, respectively. Box size indicates the length of motifs.

In addition, conserved motifs could also play a part in the function of SPLs [4,29], although the significance of these motifs needs to be further investigated. Twenty motifs were identified (Figure 5 and Table S3). The number of motifs in each SPL varied from 1 to 14 (Figure 5). Motif 1 existed in all SPLs analyzed, which is exactly the SBP domain. Except for RoSPL3, PrpSPL12, and FvSPL13, motif 2 with SBP domain existed in almost all SPLs, and motif 9 existed in G1–G10 groups. While motifs 4, 7, 10, 13, and 15 were specific to the G10 group, motifs 14 and 17 were only in the G9 group, motifs 18 and 19 only existed in the G1 group, and motif 20 specifically existed in the G3 group. These groups with unique motifs could be valuable for specific roles of SPLs. In addition to these motifs, several motifs widely existed in more than one group, such as motif 3 found in G9, G10, and G11 groups; motif 5, 6, and 12 presented in G9 and G10 groups; motif 8 found in G1, G2, G4, and G5 groups; motif 11 found in G1, G2, and G10 groups; and motif 16 found in G3, G10, and G11 groups (Figure 5). These results indicated the conservation and diversity of SPLs. Moreover, SPLs in the same group with similar motif(s) probably have a similar biological function in the growth and development of plants. The specific and common motifs of SPLs indicated that their functions were diversified and conserved.

### 3.4 Prediction of *SPLs* targeted by miR156 in Rosaceae

Some *SPLs* in *Arabidopsis* have been confirmed to have the complementary sites of miR156 in the coding regions or 3′-UTRs of *AtSPLs*, such as *AtSPL2*, *AtSPL3*, *AtSPL4*, *AtSPL5*, *AtSPL6*, *AtSPL9*, *AtSPL10*, *AtSPL11*, *AtSPL13*, and *AtSPL15* [3,18,30]. To understand miR156-driven posttranscriptional regulation of *SPLs* in the five species analyzed from Rosaceae family, the coding region of all the 92 *SPLs* was searched for the targets of miR156. Totally, 48 *SPLs* were found for the potential targets of miR156, including 5 *FvSPLs*, 15 *MdSBPs*, 9 *PrpSPLs*, 8 *RoSPLs*, and 11 *PpSPLs* (Tables S4 and S5), and these genes were found to be clustered into G1, G2, G3, G4, G5, and G8 groups (Figure 1), indicating that the posttranscriptional regulation of *SPLs* by miR156 is conserved in analyzed Rosaceae species. Previous study showed that there were nine *FvSPLs* regulated by miR156, such as *FvSPL1*, *FvSPL2*, *FvSPL6*, *FvSPL7*, *FvSPL8*, *FvSPL10*, *FvSPL11*, *FvSPL12*, and *FvSPL13*, of which the locations of targets regulated by miR156 were in 3′-UTRs of *FvSPL1*, *FvSPL2*, and *FvSPL11*, and the aa length of *FvSPL13* is different from ours [23]. Here only the coding regions of all *SPL* genes were used; therefore, only five *FvSPLs* targeted by miR156 were obtained. The number of target *SPLs* of miR156 in apple and pear confirmed the previous reports [9,10]. Here the nine *PrpSPLs* in peach and eight *RoSPLs* in black raspberry as the potential targets of miR156 were reported for the first time.

### 3.5 Function analysis of *SPL* genes in Rosaceae

**Figure 6:**
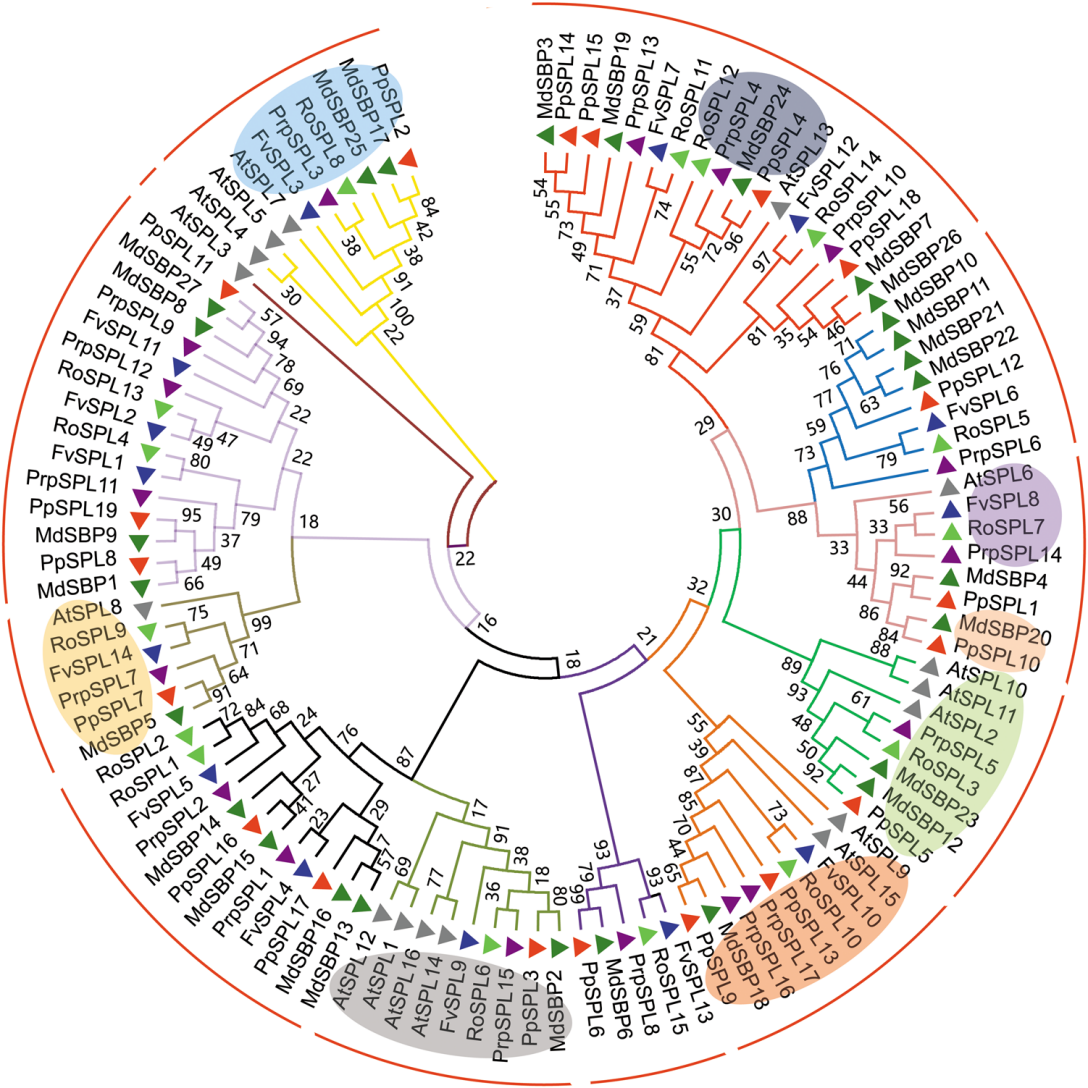
Phylogenetic tree of SPL protein from *Arabidopsis*, strawberry, apple, peach, black raspberry, and pear. During plant evolution, in different species, genes with similar functions are usually strongly related to each other and are on the same branch in a phylogenetic analysis [44]. Therefore, the functions of unknown genes could be predicted from known genes based on the phylogenetic analysis. The functions of some *SPL* genes have been studied in the model plant *A. thaliana*. Here according to the phylogenetic relationship of *SPL* genes from the five studied Rosaceae species and *A. thaliana*, the roles of the some *SPLs* could be inferred through *AtSPLs*. Proteins with similar functions are indicated with colors.

Phylogenetic relationship is very useful in elucidating the evolution and function divergence of the *SPL* gene family. In *Arabidopsis*, the function of some *SPLs* has been proved. Therefore, according to the phylogenetic relationship of *SPLs* in *Arabidopsis* and the five species from Rosaceae family, we infer the function of some *SPL* genes in Rosaceae. Earlier studies have shown that *AtSPL9* and *AtSPL15* promote the phase transition from juvenile to adult [31,32], influence the development of abaxial trichomes on adult leaves [33], and regulate the flowering of plants under right conditions [31]. Here *FvSPL10*, *RoSPL10*, *PpSPL13*, *PrpSPL16*, *PrpSPL17*, *MdSBP18*, and *PpSPL9* were clustered closely with *AtSPL9* and *AtSPL15* (Figure 6). Therefore, these genes may have the potential roles in phase transition from juvenile to adult, trichome development, and flowering time. In addition, *PpSPL10* and *PpSPL13* could interact with *PpMYB10*, which was significantly increased in the biosynthesis of anthocyanin in different developmental stages in Chinese sand pear [34], and *PpSPL10* could form a complex with *PpMYB10* [21]. *MdSBP20* and *PpSPL10* were clustered in a small branch of the phylogenetic tree (Figure 6), therefore, they may play a part in regulating anthocyanin biosynthesis. *AtSPL13* also contributes to both the transition from juvenile to adult and from vegetative to reproductive [35]. Here *MdSBP24*, *PpSPL4*, *PrpSPL4*, and *RoSPL12* with *AtSPL13* belonged to the homologous group (Figure 6). Therefore, these *SPLs* have the potential roles in the transition from juvenile to adult and from vegetative to reproductive. *AtSPL14* was previously reported to be involved in sensitivity to fumonisin B1 associated with programmed cell death and plant architecture development [14]. *AtSPL1* and *AtSPL12* confer plant thermotolerance at the reproductive stage [36]. *PpSPL3*, *MdSBP2*, *PrpSPL15*, *RoSPL6*, and *FvSPL9* were clustered into one group with *AtSPL14*, *AtSPL16*, *AtSPL1*, and *AtSPL12* (Figure 6), which may have the function similar to *AtSPL14*, *AtSPL1*, and *AtSPL12*. *AtSPL2*, *AtSPL10*, and *AtSPL11* play a role in morphological change related to shoot maturation in the reproductive phase [37]. *PpSPL5*, *MdSBP12*, *MdSBP23*, *RoSPL3*, and *PrpSPL5* with *AtSPL2*, *AtSPL10*, and *AtSPL11* belonged to one group (Figure 6), indicating that these *SPLs* may have similar function. *AtSPL6* has a function in resisting the bacterial pathogen *Pseudomonas* syringae expressing the AvrRps4 effector, and positively regulates defense gene expression [38]. *FvSPL8*, *RoSPL7*, and *PrpSPL14* were clustered closely with *AtSPL6* (Figure 6), indicating that these genes may have the role in defense against pathogens. *AtSPL7* is a regulator of Cu homeostasis in *Arabidopsis*, which can bind directly to the Cu-response element (CuRE) with a core sequence of GTAC [15]. *MdSBP25*, *PpSPL2*, *MdSBP17*, *RoSPL8*, *PrpSPL3*, *FvSPL3*, and *AtSPL7* belonged to the same group (Figure 6), indicating that these *SPLs* may regulate Cu homeostasis. *AtSPL8* regulates the development of pollen sac [39], fertility [40], biosynthesis, and signaling of GA [41]. *RoSPL9*, *PpSPL7*, *FvSPL14*, *MdSBP5*, and *PrpSPL7* were clustered into the same group with *AtSPL8* (Figure 6). Therefore, these *SPLs* may be involved in the development of pollen sac, male fertility, biosynthesis, and signaling of GA.

## 4 Discussion

SPLs are the specific transcription factors in plant, which contain the very conservative SBP domain. *SPL* genes are very important for plant development, signaling, and defense mechanisms, which have been reported in many plants, such as *Arabidopsis*, rice, maize, and tomato. However, comprehensive molecular evolutionary and function analysis remain elusive. The Rosaceae family has significant economic value, including the specialty fruit crops. Because of its potential health benefits, black raspberry (*R. occidentalis* L.) is a famous fruit crop [42]. The draft genome of black raspberry with 243 Mb has been recently published [43]. However, compared with other species from Rosaceae family, such as strawberry, apple, and pear, the progress of biological research on black raspberry is much slower, and the function of *SPLs* in black raspberry is almost unknown. Through a genome-wide identification, we obtained the set of *RoSPLs* for the first time (Table 1), showing at least 15 *RoSPLs* in *R. occidentalis*. Furthermore, we systematically analyzed the 92 *SPL* family genes in strawberry, apple, peach, black raspberry, and pear, including conservative domains and motifs, phylogenetic relationships, posttranscriptional regulation, and physiological function.

Phylogenetic analysis of genes is regarded as a very important basis for studying gene functions. During plant evolution, in different species, genes with similar functions are usually strongly related to each other and are on the same branch in a phylogenetic analysis [44]. Therefore, phylogenetic relationship could provide a convenient base for the identification of gene function. For example, the roles of some *GSKs* in cotton fiber development and stress responses were demonstrated through whole-genome characterization and phylogenetic analysis [45], and the LsToll-13 was clustered with the TLR13 by phylogenetic analysis and might be involved in the immune response of *L. striatellus* to RSV infection [46]. The analysis of phylogenetic relationship is very important step to discover the evolution and function divergence of the *SPLs*. Owing to the publication of more and more plant genome sequences, phylogenetic analysis of *SPL* genes at genome scale is receiving attention. For example, Li et al. [47] identified *SPL* genes from cassava, rubber tree, physic nut, and castor bean and carried out phylogenetic analysis for these genes. Zhang et al. [44] investigated the evolutionary relationships and divergence of *SPL* genes from moss, *Arabidopsis*, rice, and maize. Here, based on the conservative domains and motifs, phylogenetic analysis of the 92 full-length proteins showed that *SPL* gene family in five studied plant species was distributed into 11 groups. SPLs from different plant species in the same group shared common ancestors, some *FvSPLs* may be lost or may be developed in Rosaceae species, and there was a closer relationship between apple and pear and strawberry and black raspberry, respectively. Genes within the same group indicated their functional redundancy (Figure 1). Compared to domain sequences, the full-length proteins provide more information and more reliable evidence for SBP-box gene family. To illustrate the function of *SPLs* in five studied Rosaceae species, phylogenetic analysis of the full-length proteins of 108 SBP-box genes from strawberry, apple, peach, black raspberry, pear, and *Arabidopsis* was carried out (Figure 6). Compared with the Abdullah’s study on strawberry, pear, peach, Mei, and *Arabidopsis* [11], we’ve excluded more groups. This result is consistent with the phylogenetic analyses of strawberry, apple, peach, black raspberry, and pear except for *AtSPL3*. Based on the phylogenetic relationships between the five Rosaceae species and *Arabidopsis*, we predicted that *FvSPL10*, *RoSPL10*, *PpSPL13*, *PrpSPL16*, *PrpSPL17*, *MdSBP18*, and *PpSPL9* may regulate plant growth and development; *PpSPL10* and *MdSBP20* may involve in anthocyanin biosynthesis; *MdSBP24*, *PpSPL4*, *PrpSPL4*, and *RoSPL12* may play a role in the vegetative-to-reproductive transition; *PpSPL3*, *MdSBP2*, *PrpSPL15*, *RoSPL6*, and *FvSPL9* may have significant roles in sensitivity to abiotic factors; *PpSPL5*, *MdSBP12*, *MdSBP23*, *RoSPL3*, and *PrpSPL5* may control morphological change in the reproductive phase; *FvSPL8*, *RoSPL7*, and *PrpSPL14* may play a role in defense against pathogens; *MdSBP25*, *PpSPL2*, *MdSBP17*, *RoSPL8*, *PrpSPL3*, and *FvSPL3* may regulate Cu homeostasis; and *RoSPL9*, *PpSPL7*, *FvSPL14*, *MdSBP5*, and *PrpSPL7* may involve in the development of pollen sac, male fertility, biosynthesis, and signaling of GA. It provides a basic understanding necessary for the future research on the function of *SPL* genes in Rosaceae species.

Up to this point, many microRNAs have been found in plants, and their roles in gene expression have been verified. miRNAs can clear their target mRNAs or repress translation by binding to their target mRNAs and forming RNA-induced silencing complex to regulate plant growth, development, metabolism, and abiotic and biotic stress responses. miR156 is one of the very conserved microRNA families in plants [20], and miR156-SPL modules have been believed to have a very important effect on the transition from juvenile to adult, flowering time, roots, shoots, leaves and fruit development, fertility, secondary metabolism, and stress responses [18,19,20,21,31,40,48,49,50,51]. Previous studies showed that more than half of *SPLs* were predicted to be miR156 mediated in certain species. For example, among the 16 *AtSPLs* in *Arabidopsis*, 10 are the targets of miR156 [3,18,30]. In rice, 11 of 19 *OsSPLs* contain complementary sequences to OsmiR156 [4]. In tomato, 15 *SPLs* were found, 10 of which have target sites of miR156 [6]. Among the five studied Rosaceae species, 11 of 19 *PpSPLs* were the potential miR156 targets in pear [10], 15 of 27 *MdSBP* genes were the complementary sequences to the miR156 in apple [9], and 9 of 14 *FvSPL* genes were the potential targets of miR156 in strawberry [23]. Here we predicted that 5 *FvSPLs*, 11 *PpSPLs*, and 15 *MdSBPs* were the potential targets of miR156 in their coding region, which supported the previous studies. At the same time, eight *RoSPLs* in black raspberry and nine *PrpSPLs* in peach targeted by miR156 were predicted for the first time. It indicated that miR156 might be involved in anthocyanin biosynthesis, vegetative transition from juvenile to adult, morphological change in the reproductive phase, and defense against pathogens based on the potential function of *SPLs* in the five studied Rosaceae species. These results enlarge the regulatory network of *SPLs* and help to further study the regulatory mechanism of *SPL* family genes in Rosaceae species.

## 5 Conclusions

Among the 92 *SPLs* from the five studied Rosaceae species, 15 *RoSPLs* in *R. occidentalis* were identified for the first time, and some *FvSPLs* may be lost or developed. Forty-eight *SPLs* have complementary sequences to miR156, of which nine *PrpSPLs* in peach and eight *RoSPLs* in black raspberry were reported for the first time as the potential targets of miR156, suggesting the conserved regulatory effects of miR156 on *SPLs* and indicating the roles of miR156-SPL modules in plant growth, development, and defense responses. Further studies are necessary to discover the possible roles and the regulatory mechanism of *SPLs* in Rosaceae species.
